# Long-Term Survival After Radical Resection of Rectal Cancer With Synchronous Solitary Rib Metastasis: A Case Report

**DOI:** 10.7759/cureus.92025

**Published:** 2025-09-10

**Authors:** Hiroyuki Hazama, Kazumasa Nakamura, Kohei Koido, Takeshi Oshima, Kou Ohata

**Affiliations:** 1 Gastrointestinal Surgery, Shizuoka General Hospital, Shizuoka, JPN

**Keywords:** bevacizumab, bone metastasis, capox, case report, colorectal cancer, rectal cancer, rib metastasis

## Abstract

Synchronous bone metastasis from rectal cancer is exceedingly uncommon, typically associated with poor prognosis, and lacks a standardized treatment strategy. We report the case of a 67-year-old woman who was asymptomatic and referred for evaluation after a positive fecal occult blood test at screening. Staging work-up revealed rectal cancer with a solitary synchronous metastasis in the left fourth rib. The rib tumor was resected with chest wall reconstruction for both diagnostic confirmation and local control, followed 52 days later by robot-assisted low anterior resection of the primary lesion. R0 resection was achieved for both sites. Considering the risk of occult systemic disease, adjuvant chemotherapy with capecitabine plus oxaliplatin (CAPOX) and bevacizumab was initiated, subsequently switched to capecitabine plus bevacizumab, and continued for two years. The patient remains disease-free five years after primary tumor resection. This case suggests that, in carefully selected patients with solitary synchronous bone metastasis from rectal cancer, combined radical resection and systemic therapy may provide long-term disease control and survival benefit.

## Introduction

Bone metastasis from colorectal cancer (CRC) is uncommon compared with liver or lung metastases, and synchronous bone metastasis at the time of diagnosis is even more infrequent, usually portending a poor prognosis [1,2]. Solitary bone metastasis without involvement of other organs is particularly rare, and clinical evidence to guide decision-making remains limited [3]. Consequently, the role of curative-intent local treatment - such as primary tumor resection, metastasectomy, or ablation - for bone metastasis has not been firmly established. Recent reports, however, have suggested that long-term survival may be achievable in highly selected cases through a multidisciplinary approach that combines R0 resection of both the primary and metastatic lesions with systemic therapy [4,5]. Furthermore, long-term survival after resection of liver and lung metastases is well established [6-8].

## Case presentation

A 67-year-old woman, with no remarkable symptoms, was referred to the gastroenterology department after a positive fecal occult blood test was detected during screening. She was diagnosed with rectal cancer and a synchronous solitary bone metastasis in the left fourth rib (cT1N1aM1b (bone), Stage IVB, TNM Classification of Malignant Tumours (TNM UICC) 8th edition) and was subsequently referred to our department.

The patient's family history includes the father with pancreatic cancer and the mother with gastric cancer. Her medical history includes mastopathy at age 45 years, hypertension and cerebral aneurysm surgery at 56 years, hyperlipidemia at 57 years, diabetes mellitus at 63 years, and atrial fibrillation at 67 years.

Initial findings

On admission, she was alert, with stable vital signs and no abnormal findings on physical examination. Her Eastern Cooperative Oncology Group (ECOG) performance status was 0.

Laboratory data

No abnormalities were observed in hematological or biochemical tests. Regarding tumor markers, carcinoembryonic antigen (CEA) was elevated at 25.5 ng/mL, while carbohydrate antigen 19-9 (CA19-9) was within the normal range.

Staging work-up

Colonoscopy

**Figure 1:**
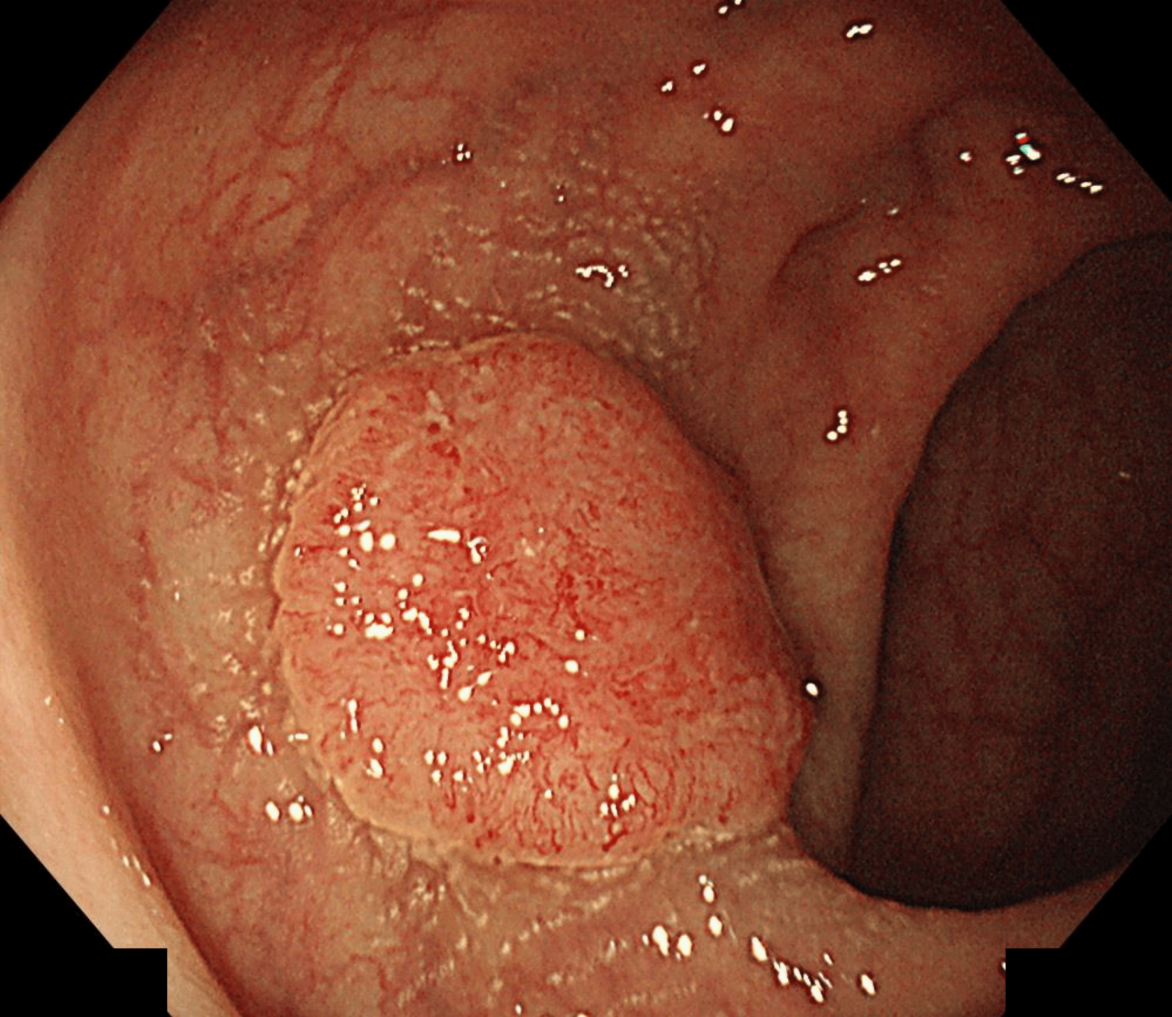
White-light colonoscopy showing a 0-IIa+IIc lesion located 7 cm from the anal verge.

**Figure 2:**
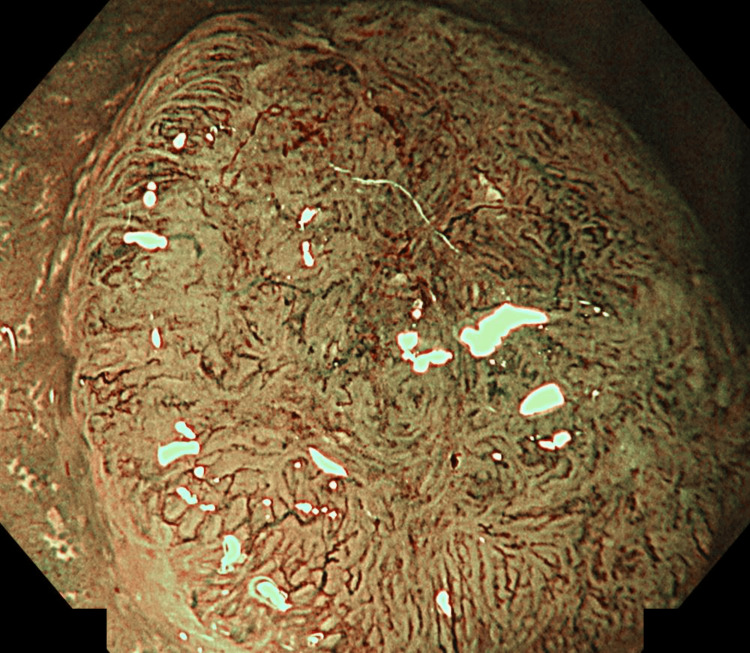
Magnifying NBI image demonstrating a type 3 lesion according to the JNET classification, consistent with deep submucosal invasion. JNET: Japan NBI Expert Team

**Figure 3:**
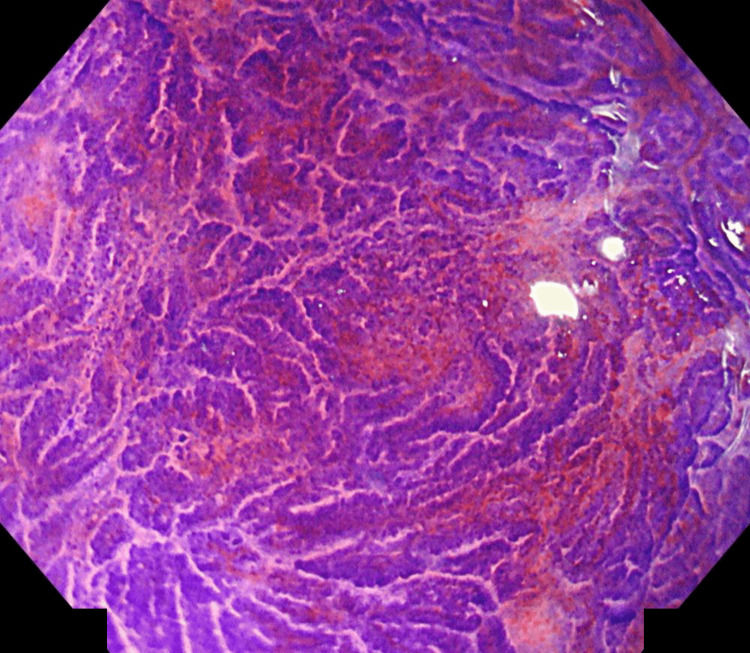
Pit pattern analysis using chromoendoscopy showing Kudo’s type VI (irregular) and focal VN (non-structured).

A 15-mm superficial elevated lesion with central depression was identified 7 cm from the anal verge, classified as 0-IIa+IIc according to the Paris classification (Figure 1). Magnifying narrow-band imaging demonstrated a type 3 lesion by the Japan NBI Expert Team (JNET) classification, consistent with deep submucosal invasion (Figure 2). Pit pattern analysis revealed Kudo’s type VI (irregular) predominantly and partially VN (non-structured) (Figure 3). A biopsy confirmed a well-differentiated adenocarcinoma.

Contrast-Enhanced CT

**Figure 4:**
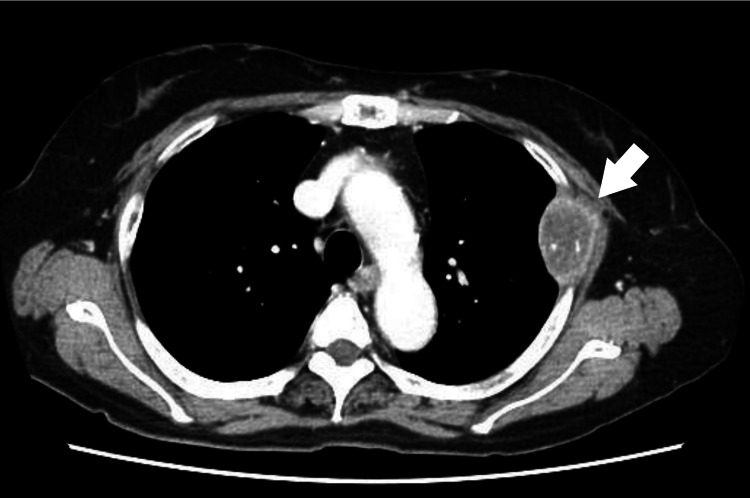
CT showing a destructive mass in the left fourth rib with ring-like enhancement (arrow).

A 7-mm perirectal lymph node was noted. A destructive mass, 48 × 28 mm in size, was detected in the left fourth rib with ring-like enhancement, suspicious for bone metastasis (Figure 4).

Fluorodeoxyglucose (FDG)-PET/CT

**Figure 5:**
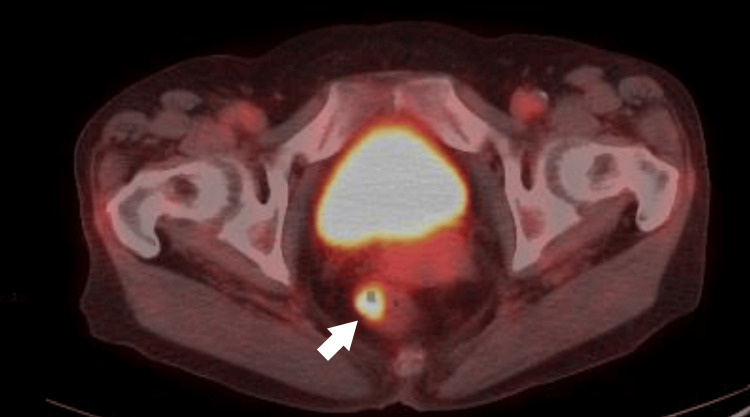
Fluorodeoxyglucose (FDG)-PET/CT image showing intense uptake in the rectal lesion (SUVmax: 10.59, arrow).

**Figure 6:**
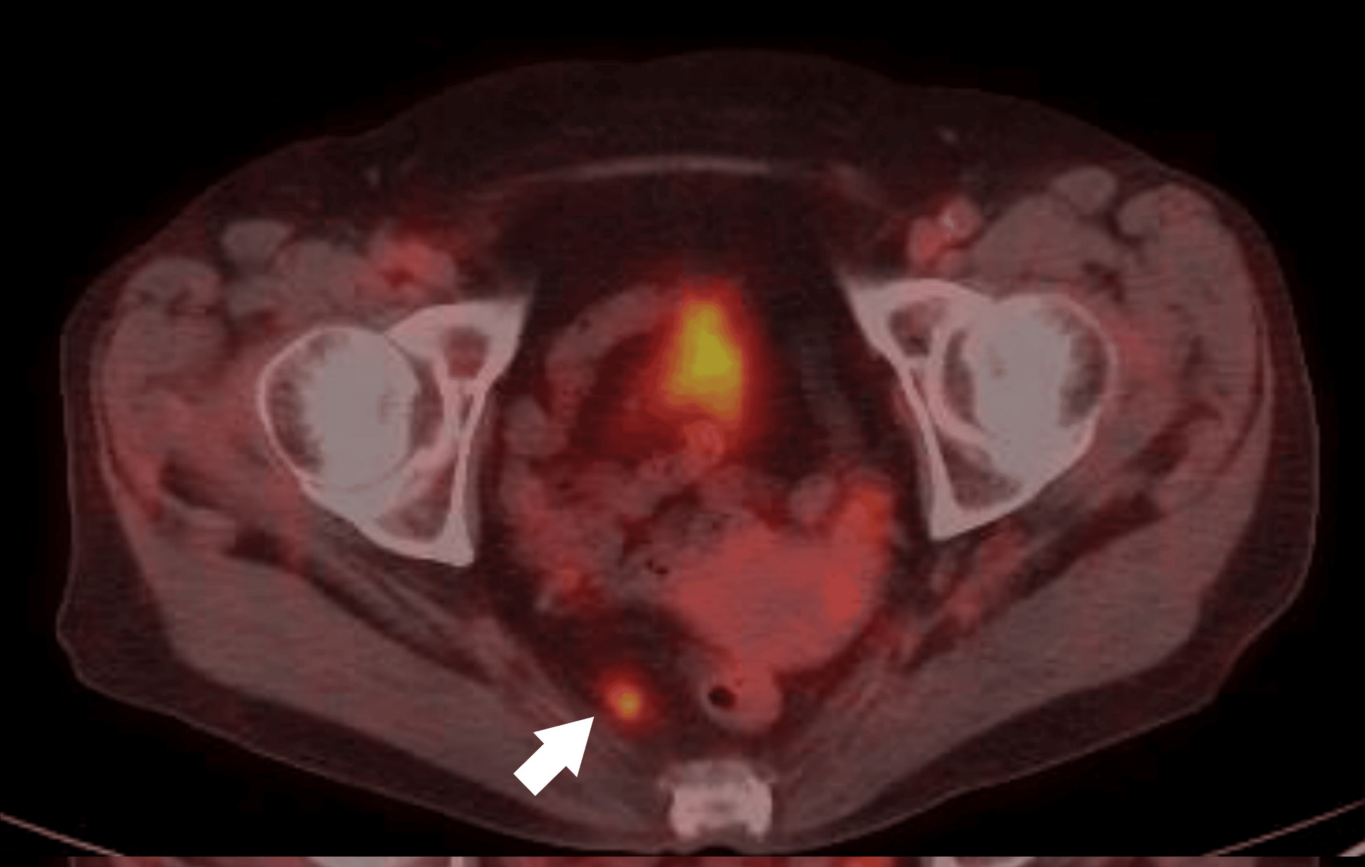
Fluorodeoxyglucose (FDG)-PET/CT image showing uptake in a perirectal lymph node (SUVmax: 3.67, arrow).

**Figure 7:**
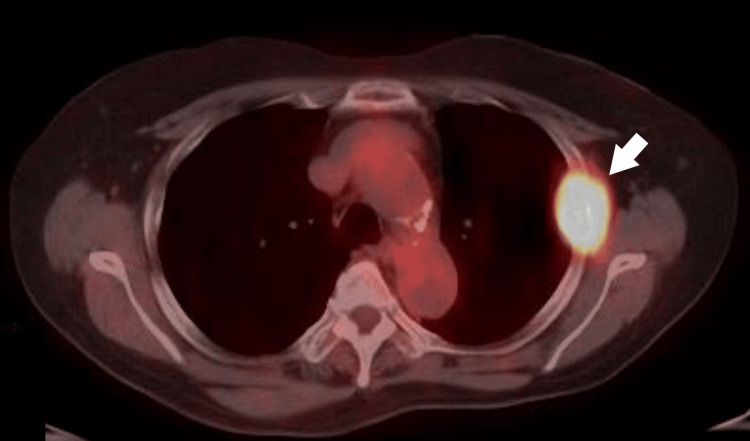
Fluorodeoxyglucose (FDG)-PET/CT image showing uptake in the rib lesion (SUVmax: 15.16, arrow).

Intense uptake was observed in the rectal lesion (SUVmax: 10.59) (Figure 5), in a perirectal lymph node (SUVmax: 3.67) (Figure 6), and in the left fourth rib lesion (SUVmax: 15.16) (Figure 7).

Molecular findings

Molecular analysis revealed a RAS mutation, while mismatch repair protein expression was preserved (proficient mismatch repair, pMMR).

Treatment strategy

Given her good performance status and the resectability of both lesions, the treatment plan was to resect the rib metastasis first for diagnostic confirmation and local control, followed by resection of the primary rectal lesion.

Surgery (Rib Metastasis)

**Figure 8:**
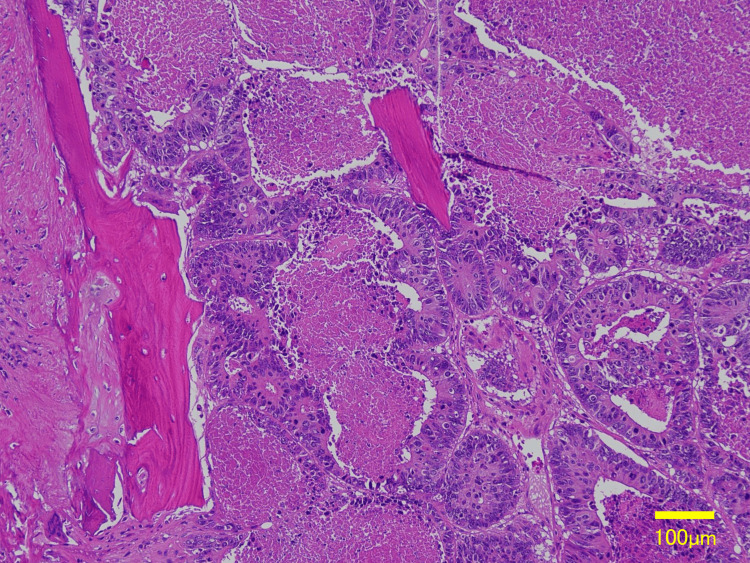
Histopathology (H&E) demonstrating moderately to well-differentiated adenocarcinoma with central necrosis. Scale bar = 100 µm

En bloc resection of the rib tumor, including adjacent chest wall structures, was performed with prosthetic chest wall reconstruction. Pathology showed invasive proliferation of moderately to well-differentiated adenocarcinoma with central necrosis (Figure 8).

**Figure 9:**
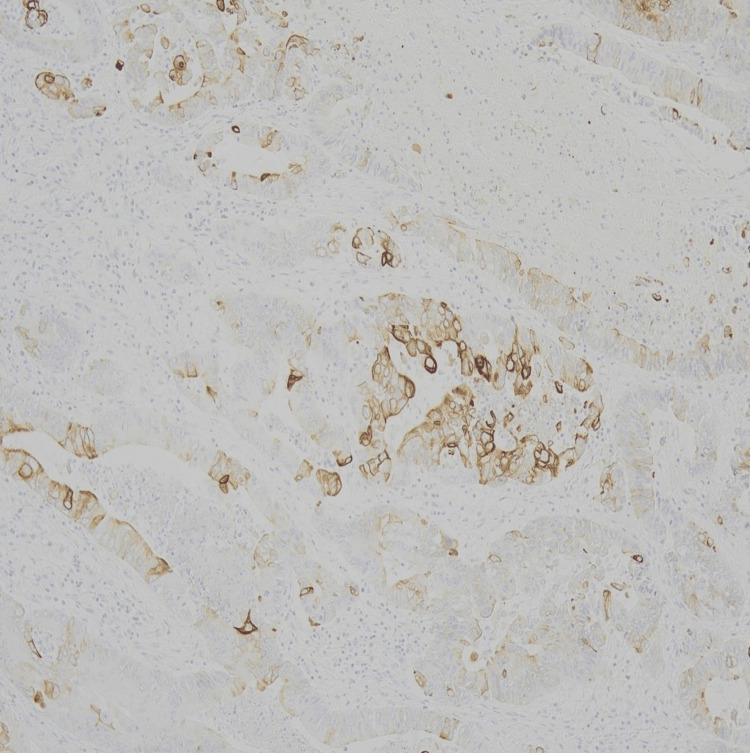
Immunohistochemistry for CK20 showing cytoplasmic positivity, supporting colorectal origin.

**Figure 10:**
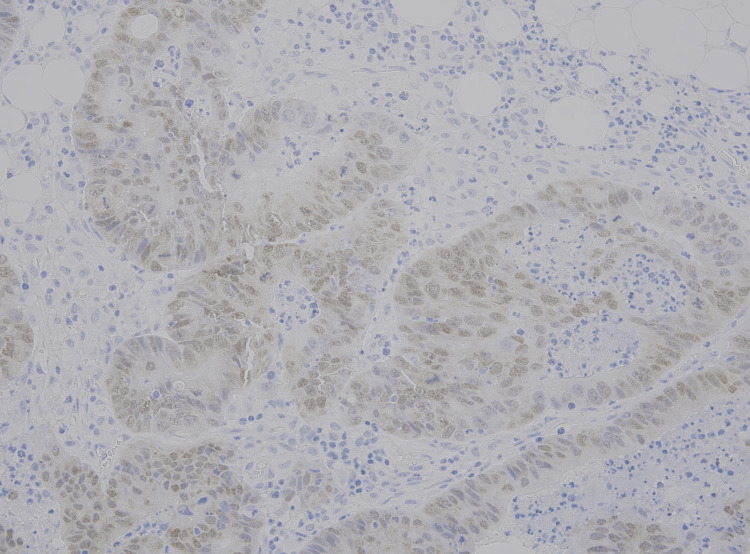
Immunohistochemistry for CDX2 showing nuclear positivity, supporting the colorectal origin.

Immunohistochemistry (CK20-positive, CK7 and TTF-1-negative, weak CDX2-positive) supported the colorectal origin (Figures 9-10).

Surgical margins were negative (R0).

Surgery (Rectal Lesion)

Fifty-two days later, robot-assisted low anterior resection with D3 lymphadenectomy was performed. No hepatic or peritoneal metastases were observed intraoperatively.

Pathology (Rectum)

**Figure 11:**
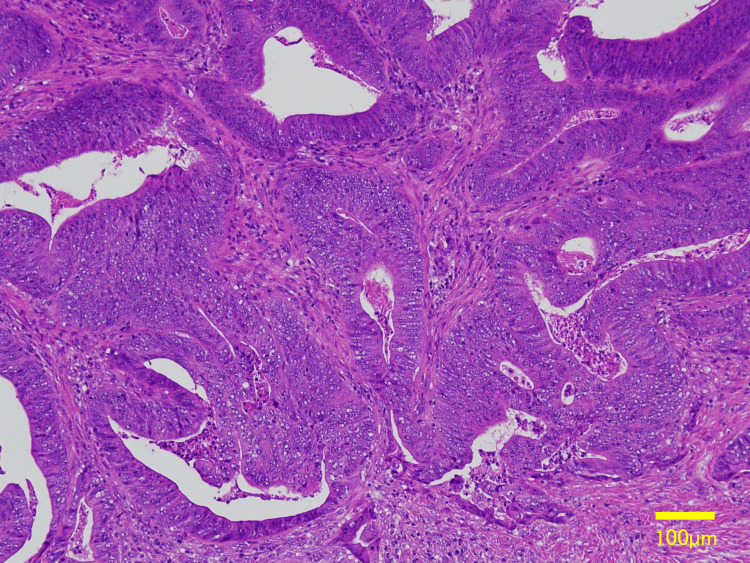
Histopathology (H&E) demonstrating an invasive moderately differentiated adenocarcinoma. Scale bar = 100 µm

The resected rectal lesion was located in the Rb segment, measuring 1.5 × 1.4 cm, and classified as 0-IIa+IIc. Histology demonstrated an invasive moderately differentiated adenocarcinoma, morphologically consistent with the rib metastasis (Figure 11). According to the UICC TNM classification (8th edition), the tumor was staged as pT1, pN1a, and M1b (bone metastasis), corresponding to Stage IVB. All resection margins were negative (R0).

Postoperative Course and Systemic Therapy

The postoperative course was uneventful, and the patient was discharged on postoperative day six after rectal surgery. Considering the high risk of recurrence, systemic chemotherapy was initiated in line with guidelines for advanced CRC. She received CAPOX plus bevacizumab for 10 cycles, followed by maintenance capecitabine plus bevacizumab, for a total of two years. She has remained recurrence-free for five years following rectal resection.

## Discussion

This case represents a rare presentation of rectal cancer with a synchronous solitary rib metastasis, in which curative resection of both the primary lesion and the metastatic site was performed, followed by two years of systemic chemotherapy, resulting in long-term recurrence-free survival.

Bone metastasis from CRC has been reported in approximately 6-10% of cases [1,2]. In most patients, bone metastases coexist with liver and/or lung metastases, while isolated bone metastasis is exceedingly rare. Population-based data have demonstrated that rectal cancer is more likely than colon cancer to develop bone and lung metastases [9], which is consistent with the present case.

Recent prognostic analyses have also highlighted the poor outlook of CRC patients with bone metastases overall. In a Surveillance, Epidemiology, and End Results (SEER)-based study of 178 institutional and 1,124 registry cases, lymph node status, primary tumor sidedness, and the site of bone metastasis were identified as independent prognostic factors. Right-sided CRC with bone metastases was associated with significantly worse survival, while node-negative disease had relatively favorable outcomes [3]. In our case, despite the presence of nodal involvement (pN1a), complete resection of both lesions followed by prolonged systemic therapy may have contributed to overcoming this unfavorable prognostic factor.

More recent case reports have described long-term survival following resection of solitary bone metastases [4,5], suggesting that surgery may be a reasonable option in selected patients. Furthermore, long-term survival after resection of liver and lung metastases is well established [6-8], lending indirect support to the role of metastasectomy in bone metastasis when technically feasible.

Another important consideration is that bone metastasis likely reflects systemic disease, and micrometastases in other organs may already exist at the time of diagnosis [9]. Local treatment alone may therefore be insufficient. In this case, systemic chemotherapy with CAPOX plus bevacizumab was administered for two years postoperatively. Although there is no established evidence supporting such prolonged therapy after bone metastasectomy, by contrast, in Stage III colorectal cancer, a six‑month course of adjuvant FOLFOX or CAPOX is considered standard of care [10]. Analogous studies in colorectal liver metastases suggest potential benefit: for instance, the HEPATICA trial evaluated CAPOX with or without bevacizumab as adjuvant therapy after hepatic metastasectomy, showing a trend toward improved disease‑free survival, although early termination limited statistical power [11]. Moreover, systematic reviews in this setting have demonstrated improvement in relapse‑free survival but an inconsistent impact on overall survival [12]. Thus, extended systemic therapy in this patient may have helped suppress occult micrometastatic disease and contributed to her favorable outcome.

Limitations

This is a single case report, and the benefit of prolonged systemic therapy after bone metastasectomy remains speculative. Larger case series or prospective studies are needed to establish optimal treatment strategies in this setting.

## Conclusions

Radical resection can be a feasible treatment option for synchronous solitary bone metastasis from rectal cancer in carefully selected patients. Combined with systemic chemotherapy, this approach may suppress micrometastases and provide the opportunity for long-term disease-free survival. Further accumulation of similar cases is warranted to clarify the role of surgery and systemic therapy in this setting.
